# Using next generation antimicrobials to target the mechanisms of infection

**DOI:** 10.1038/s44259-023-00011-6

**Published:** 2023-09-22

**Authors:** Kavita Gadar, Ronan R. McCarthy

**Affiliations:** Division of Biosciences, Department of Life Sciences, College of Health, Medicine and Life Sciences, Brunel University London, Uxbridge, UB8 3PH United Kingdom

**Keywords:** Antimicrobials, Bacteria

## Abstract

The remarkable impact of antibiotics on human health is being eroded at an alarming rate by the emergence of multidrug resistant pathogens. There is a recognised consensus that new strategies to tackle infection are urgently needed to limit the devasting impact of antibiotic resistance on our global healthcare infrastructure. Next generation antimicrobials (NGAs) are compounds that target bacterial virulence factors to disrupt pathogenic potential without impacting bacterial viability. By disabling the key virulence factors required to establish and maintain infection, NGAs make pathogens more vulnerable to clearance by the immune system and can potentially render them more susceptible to traditional antibiotics. In this review, we discuss the developing field of NGAs and how advancements in this area could offer a viable standalone alternative to traditional antibiotics or an effective means to prolong antibiotic efficacy when used in combination.

## Introduction

In the early 1900s, infectious diseases were the leading cause of death across almost every age demographic worldwide^1–4^. However, during the 20th century there was a dramatic decline in the number of people dying from infectious diseases. This decline can be at least in part, attributed to the advent of antibiotics spearheaded by Sir Alexander Fleming’s discovery of penicillin^2,5^. The term ‘antibiotic’ was first described in 1941, by Prof Selman Waksman, as a small molecule produced by a microbe that possesses antagonistic properties against the growth of other microbes^6^. Antibiotics work by inhibiting the growth of bacteria (bacteriostatic) or by killing the bacteria (bactericidal)^7^. Their mechanisms of action vary but they typically target essential bacterial functions such as transcription, translation, cell wall synthesis and DNA replication. Targeting such essential processes imposes a strong negative selection pressure upon bacteria, driving the evolution of antibiotic resistance^8^. This has meant that the efficacy of frontline antibiotics is being eroded continually by the spread of transmissible resistance conferring genetic elements and the evolution of multi-drug resistant (MDR) pathogens. This has led to the antibiotic resistance crisis, a major threat to our global healthcare infrastructure and modern medicine. With respect to mortality, the scale of this underreported crisis is akin to other major threats facing humanity such as the climate emergency, with 4.95 million deaths associated with bacterial antimicrobial resistant (AMR) infections in 2019, compared to 5.08 million deaths due to climate change^9,10^. Worryingly, there is an emerging body of compelling evidence that climate change is exacerbating the AMR crisis, with an increased regional ambient temperature being associated with a higher prevalence of antibiotic resistance^11,12^.

Our current systems and infrastructure for the clinical development of antibiotics and their transition from the bench to the bedside is failing with an exponential decline in the number of newly developed and approved antibiotics over the last three decades^13^. The significant costs and time associated with bringing a new class of antibiotic to the market and their lack of financial return has disincentivised the pharmaceutical industry. As a result, most multi-national pharmaceutical companies have shelved their antibiotic development pipelines over the last two decades and many start-ups have folded under these significant pressures. This maelstrom of exits has created a major vulnerability in our healthcare infrastructure driving alarming increases in the number of deaths associated with antibiotic-resistant infections^9^. The financial burdens associated with treating antibiotic-resistant infections is also a major consideration with the estimated medical cost of one patient with an antibiotic-resistant infection in the US ranging from $18,588 to $29,069^14^. With the increasing rates of AMR, it is predicted that the annual cost of AMR could rise to $100 trillion by 2050^15^. This is forcing a global rethink of how we bring new antibiotics to market and driving more research into the exploration of alternatives to traditional antibiotics such as phage, vaccines and virulence targeting next-generation antimicrobials (NGAs). Additionally, the repurposing of existing drugs as anti-virulence treatments has gained momentum, providing rapid development with a lower cost, and expanding the range of potential combination therapy options.

**Fig. 1:**
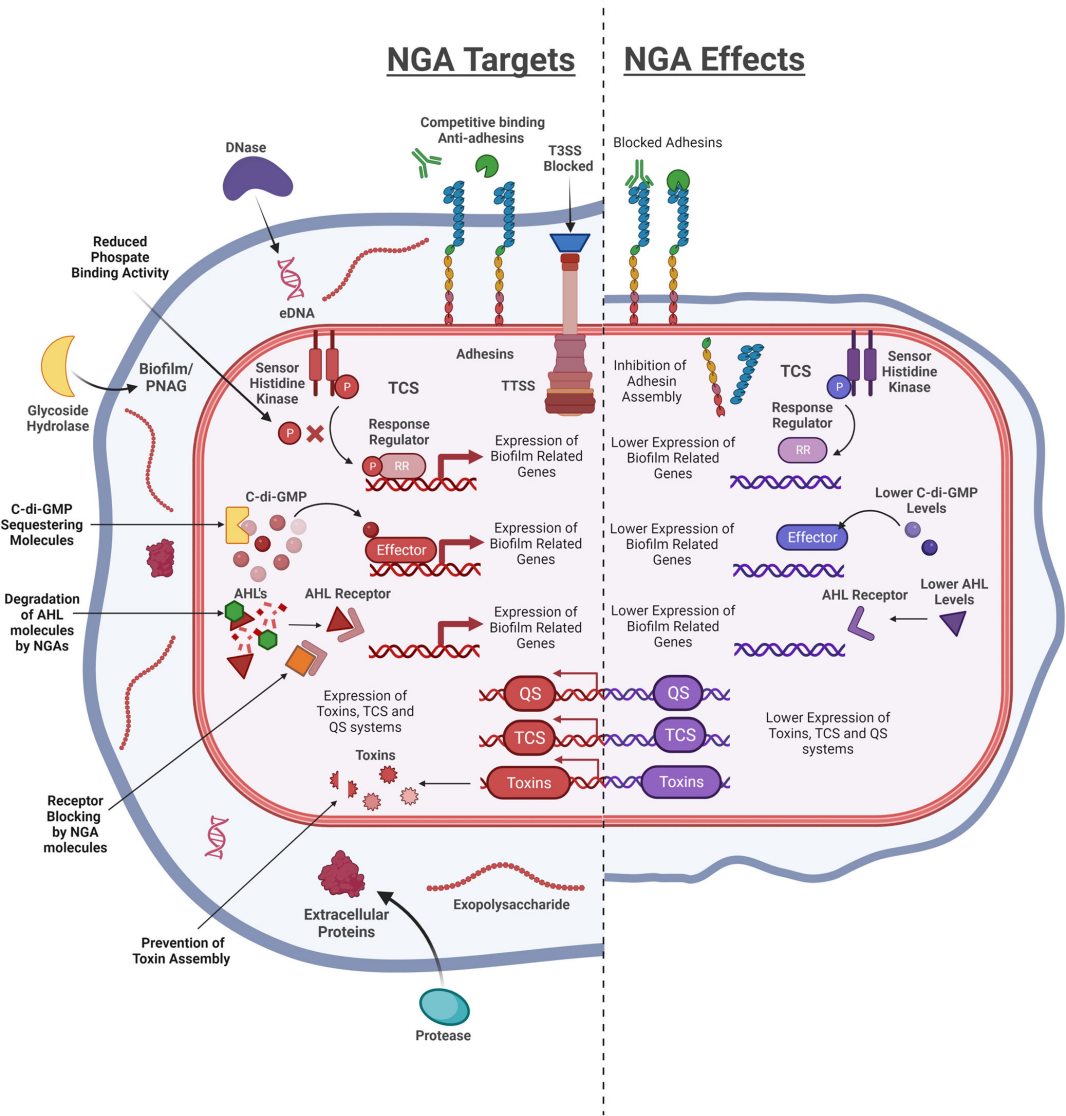
A summary of the different cellular and extracellular targets of NGA (Left) and the impact on different virulent phenotypes (Right).

NGAs are compounds that have antivirulence properties at concentrations that do not impact bacterial viability, therefore minimising the selective pressure they apply and the probability of resistance evolution. The primary function of virulence factors in an infection context is to allow the pathogen to colonise the host^16^. Thus, targeting virulence factors disrupts the pathogenic potential of these bacteria making it more difficult for them to colonise the host, making them more vulnerable to clearance by the immune system and potentially rendering them more susceptible to traditional antibiotics (Fig. 1). This review discusses the developing field of NGAs and how advancements in this area could offer a viable standalone alternative to traditional antibiotic use or potentially prolong the efficacy of frontline antibiotics when administered in combination.

## Colonisation – Disrupting the structural integrity of a biofilm

Biofilms are communities of bacterial cells that adhere to each other or a surface and are encased in a matrix made up of structural components such as polysaccharides, proteins, and extracellular DNA^17^. Studies have found that ~80% of recurrent or chronic infections are attributed to the formation of bacterial biofilms, highlighting their importance in infection^18^. The formation of biofilms is a multi-step process that starts with the attachment of bacteria to a biotic or abiotic surface or their aggregation to each other. These micro-colonies then grow and expand with the recruitment of surrounding cells or aggregates and develop into larger three-dimensional community structures with complex nutrient transportation networks. As the biofilm grows, it enters the final stage of its lifecycle, where cells detach from the biofilm and may spread as planktonic cells or aggregates^19^. Growing in a biofilm provides increased protection from antibiotics, disinfectants and the host immune system. In comparison to cells in a planktonic state, bacteria embedded in biofilms display an increased tolerance to antibiotics by over 10-1000-fold due to poor penetration of antibiotics, heterogeneous transcription and the presence of persister cells^20–22^. These factors are all exacerbated in a polyspecies biofilm where additional behaviours such as cooperation between sensitive and resistant strains or species can occur^23,24^. Therefore, targeting biofilms is an attractive strategy for the development of NGAs (Table 1). The use of extracellular enzymes that can disrupt biofilms by degrading the structural components of the biofilm matrix is one of the primary strategies for biofilm dispersal. By focusing on the structural integrity of the biofilm, enzymes such as DNase I, PodA and NucB can induce forced dispersal of cells from the biofilm colony and release them into the environment in a more antibiotic susceptible planktonic form^25–35^.

**Table 1:** NGAs that target and disrupt the structural integrity of biofilm.

	NGA/Active Molecule	Organism	Mode of Action	Molecular Target	In Vivo/In Vitro	Reference
Extracellular DNA	DNase I	*Pseudomonas aeruginosa, Escherichia coli*, *Acinetobacter baumannii, Staphylococcus aureus, Enterococcus faecalis*	Cleaves the eDNA of established biofilms, decreasing biomass by altering biofilm structure	eDNA	in vitro, in vivo in murine model	^26–31^
	PodA	*Pseudomonas aeruginosa*	Prevents release of extracellular DNA in the matrix of biofilm	methyl group present in pyocyanin	in vitro	^32^
	NucB	*Bacillus licheniformis*, *Staphylococcus aureus*, *Staphylococcus epidermidis*, *Staphylococcus salivarius*, *Staphylococcus constellatus*, *Staphylococcus lugdunesis*, *Staphylococcus anginosus*, *Escherichia coli*, *Streptococcus intermedius*, *Micrococcus luteus*, *Bacillus subtilis*	Bacterial DNase that degrades established biofilms	eDNA	in vitro, in vivo in marine model	^33–35^
Extracellular Proteins	Proteinase K	*Escherichia coli, Staphylococcus aureus*, *Listeria monocytogenes*, *Staphylococcus lugdunensis and Staphylococcus heamolyticus*	Degradation of proteins in the biofilm to aid in biofilm dispersal	Biofilm-associated proteins and DNA binding proteins	in vitro	^47,48,50,51,148^
	Trypsin	*Pseudomonas aeruginosa*	Cleaves peptides in the biofilm	Biofilm peptides	in vitro	^52^
	Staphopain A (ScpA) and Staphopain B (ScpB)	*Staphylococcus aureus*	*Staphylococcal* cysteine proteases that degrade biofilm-associated proteins	Biofilm-associated proteins	in vitro	^53,54^
	Antisera	*Enterococcus faecium, Staphylococcus aureus, Klebsiella pneumoniae, Acinetobacter baumannii, Pseudomonas aeruginosa, Enterobacter spp., and Escherichia coli*	Potentiate DNase induced biofilm damage, antibiotic killing and to increase the capacity of macrophage to kill bacteria	DNABII family of proteins	in vitro	^56,58^
	*Escherichia coli* IHF	*Escherichia coli*	Potentiate DNase induced biofilm damage	DNABII family of proteins	in vitro, in vivo in chinchilla model	^56^
	Humanised monoclonal antibody	*Pseudomonas aeruginosa*, *Staphylococcus aureus*, *Burkholderia cenocepcia*, and *Moraxella catarrhalis*	Potentiate DNase induced biofilm damage	DNABII family of proteins	in vitro, in vivo in chinchilla and murine model	^58–60^
Extracellular Polysaccharides	Dispersin B	*Staphylococcus aureus, Aggregatibacter actinomycetemcomitans, Staphylococcus epidermidis, Acinetobacter baumannii, Klebsiella pneumoniae, Yersinia pestis and Pseudomonas fluorescens*	Degradation of PNAG by hydrolysing the β(1,6) glycosidic linkages	PNAG	in vitro	^30,63^
	Caspofungin	*Staphylococcus aureus*	Weaken PNAG polymerisation by inhibiting Nacetylglucosamine transferase	PNAG	in vitro, in vivo in murine model	^65^
	Hydroxamic acid	*Pseudomonas aeruginosa*	Reduces biofilm matrix	Targets the zinc ion active site in elastase enzyme, LasB	in vitro	^177^
Adhesions	Pilicides	*Escherichia, Salmonella, Yersinia, Pseudomonas, Klebsiella*, and *Haemophilus*	Prevent pilus assembly and disrupt chaperone-usher pathway	PapD and PapG	in vitro	^72^
	FN075	*Escherichia coli*	Prevents polymerisation of CsgA	CsgA	in vitro	^75,76^
	BibC6	*Escherichia coli*	Prevents polymerisation of CsgA	CsgA	in vitro	^75,76^
	LPRDA	*Staphylococcus aureus*	Inhibits sortase A	Sortase A	in vitro	^178^
	Fucosides	*Aspergillus fumigatus*	Aggregate and inhibit targeted lectins	FleA	in vitro	^179^
	T2544 Antiserum	*Salmonella enterica serovar Typhi*	Enhance the uptake and clearance of bacteria	T2544	in vitro, in vivo in murine	^86^

### Targeting extracellular DNA

Extracellular DNA (eDNA) in biofilm functions as structural scaffolding within the matrix and can also modulate aggregation and adhesion to host cells and tissues^36^. Many studies have shown that the addition of exogenous deoxyribonuclease (DNase) can inhibit biofilm formation in both Gram-negative and Gram-positive bacteria, without impacting bacterial growth^37^. DNase I cleaves biofilm-associated eDNA resulting in decreased biofilm biomass and an increased antibiotic penetration. This effect has been shown in vitro and in vivo in rat models against a wide range of pathogens including *Pseudomonas aeruginosa, Escherichia coli, Acinetobacter baumannii, Staphylococcus aureus* and *Enterococcus faecalis* highlighting the broad-spectrum versatility of this approach^26–31,38^. Indeed, recombinant DNAse I has been used therapeutically for cystic fibrosis (CF) patients for over 20 years as means to decrease the viscoelasticity of sputum slowing the rate of lung function decline. It is likely, based on in vitro data, that the DNAse is also limiting pathogen biofilm formation within the CF lung^39^. The use of DNases to treat wound biofilms is relatively underexplored in comparison but they have been shown some to disrupt established biofilms and promote healing when administered in combination with silver nanoparticles in vivo^40^. This disruption of mature biofilms is attributed to the cleavage of eDNA by DNAse, compromising the structural integrity of the biofilm, which in turn allows greater penetration of DNAase enzymes^38^. The application of DNases to chronic diabetic wounds has also been shown to promote healing, but this is thought to occur through the breakdown of neutrophils extracellular traps (NETs). However, this suggests that a DNase-based chronic wound treatment has the potential to target both host and pathogen factors that are impediments to wound healing^41,42^.

Rather than targeting the eDNA after it has been integrated into the biofilm matrix, an alternative approach is to inhibit eDNA release. Purified pyocyanin demethylase (PodA) has been shown to inhibit the pyocyanin-dependent release of eDNA into the biofilm matrix, disrupting *P. aeruginosa* biofilm formation and limiting biofilm aggregate populations^32^. This approach, however, will not overcome the eDNA that is available through both host and pathogen cell lysis, suggesting that the efficacy of these more targeted approaches may be limited in comparison to exogenous DNAse application^43^. Another factor to consider is that eDNA has been shown to be protected from DNase degradation by cationic exopolysaccharides, such as the *P. aeruginosa* polysaccharide Pel, potentially limiting therapeutic efficacy^44^.

### Targeting extracellular proteins

Extracellular proteins are major constituents of the biofilm matrix^45^. Proteins such as biofilm-associated proteins and DNA-binding proteins play a crucial role in the adhesion, scaffolding and stability of the biofilm matrix^46^. The integral role of these proteins within the biofilm matrix makes them promising candidates for the development of biofilm dispersal agents. The stable yet highly reactive protease, Proteinase K, has been shown to exhibit biofilm dispersal activity in vitro against several clinically relevant pathogens^47–51^. Trypsin, a pancreatic serine protease, was found to have a non-cytotoxic biofilm degrading effect on *P. aeruginosa*^52^. Similarly, the exogenous application of staphylococcal cysteine proteases Staphopain A (ScpA) and Staphopain B (SspB) have been shown to demonstrate biofilm dispersal abilities against established *S. aureus* biofilms^53,54^.

Targeting the immune system towards biofilms associated proteins has been shown to significantly disrupt the structural lattice of eDNA and the overall biofilm. Antisera directed towards DNABII family of proteins such as integration host factor A, IhfA, has been shown to disrupt biofilms formed by each of the high-priority ESKAPE pathogens *(Enterococcus faecium, S. aureus, Klebsiella pneumoniae, A. baumannii, P. aeruginosa, Enterobacter spp.,)* as well as numerous other clinically relevant pathogens^55,56^. This approach has also been shown to potentiate DNase induced biofilm damage, antibiotic killing and to increase the capacity of macrophages to kill bacteria^55,56^. When purified *E. coli* IHF was used as an immunogen in a chinchilla animal model, with an established biofilm-associated infection, the resultant targeted immune response led to rapid resolution of the infection^56^. This strategy has also been shown to be effective when targeting polymicrobial biofilms within CF sputum solids^57^. Humanised monoclonal antibodies directed against DNABII family of proteins have also shown remarkable efficacy to disrupt single and multispecies biofilms and to potentiate antibiotic activity^58–60^.

### Targeting extracellular polysaccharides

Secreted extracellular polysaccharides are key components of the biofilm matrix that contribute to the initial establishment and persistence of biofilms^61,62^. Many studies have demonstrated the efficacy of dispersin B, a glycoside hydrolase produced by *Actinobacillus actinomycetemcomitans*, against established biofilm of pathogens such as *S. aureus, S. epidermidis, A. baumannii, K. pneumoniae, Yersinia pestis and Pseudomonas fluorescens*. This glycoside hydrolase degrades the polysaccharide poly(1,6)-*N*-acetyl-d-glucosamine (PNAG) by hydrolysing the β(1,6) glycosidic linkages^30,63^. Dispersin B has been used in combination with DNase 1 to limit *S. aureus* skin colonisation and increase biocide sensitivity in an in vivo porcine model^64^. Similarly, caspofungin, an antifungal natural product, has been shown to weaken PNAG polymerisation by inhibiting N-acetylglucosamine transferase in *S. aureus*, resulting in the structure of the biofilm matrix becoming more susceptible to fluoroquinolones in vitro and in vivo in rat models^65^.

A key consideration with NGAs that are developed to target and disperse biofilms is their potential capacity to send the aggregates and/or planktonic cells into the local microenvironment, potentially facilitating the dissemination of the bacteria to different possible infection sites or triggering bacteraemia^25^. Therefore, their application must be carefully considered with respect to the type and location of the infection.

### Reducing adhesion

The physicochemical properties of the bacterial cell surface and the receptors that decorate it, play a key role in infection, with pili binding to host cell glycoproteins for example often initiating colonisation. Disrupting surface receptor biogenesis has been shown to lead to a decrease in bacterial adhesion to host cells and tissues^66^. These changes have been shown to occur due to misfolding or an abnormal production of chaperone-usher proteins, that are responsible for the assembly and secretion of fimbrial adhesins. The resultant inhibition of host receptor interactions and alteration in surface charge effectively limits bacterial adhesion^67–69^. This suggests that targeting the assembly of pili, such as Type 1 and P pili found in *Escherichia, Salmonella, Yersinia, Pseudomonas, Klebsiella* and *Haemophilus*, may be a promising strategy for preventing bacterial infections via adhesion inhibition^70,71^.

Small molecules called pilicides have been found to prevent pilus assembly and disrupt formation of the chaperon-usher complex by binding to the active site of the periplasmic chaperones PapD and PapG that are required for the assembly of Type 1 and P pili, and thus preventing bacterial adhesion^72–74^. Sub-inhibitory concentrations of antibiotics like ciprofloxacin and amikacin can also alter the bacterial surface, impairing adhesion to host cells^69^. Bicyclic 2-pyridones, such as FN075 and BibC6, have demonstrated inhibitory effects on the assembly of curli by preventing polymerisation of the major curli subunit protein CsgA^75–80^. Curli, which are thinner amyloid polymers compared to fimbriae, play a role in adhesion and the formation of biofilms^81^.

Exploiting carbohydrates that mimic host cell surfaces is a competition-based strategy to prevent bacterial infection, with initially pioneering work by Duguid and Gillis in the 1950s demonstrating the anti-adhesive properties of mannose when applied to *E. coli*^82^. This paved the way for the development of a vast array sugar-based inhibitors and glycomimetic compounds that act as anti-adhesives by competitively inhibiting the binding of pathogens to host cells^66,82^. Multivalent compounds with increased binding avidity and monovalent inhibitors with aglucan moieties have been shown to inhibit uropathogenic *E. coli* (UPEC) infections by targeting the adhesive subunit FimH^83,84^. 3′-chloro-4′-(α-d-mannopyranosyloxy) biphenyl-4-carbonitriler, a FimH inhibitor, has shown promising therapeutic potential in the mouse urinary tract infection model, reducing bacterial load in the bladder by almost 1000-fold 3 hours after infection while also displaying favourable pharmacokinetics, such as low toxicity and renal excretion^85^.

Anti-adhesion antibodies and vaccines are also being explored as strategies to combat bacterial infections. Various approaches have been demonstrated, including immunisation with bacterial adhesins or subunits, immunogenic peptide fragments, or DNA vaccines encoding adhesins^66^. Targetting the *Salmonella enterica serovar Typhi* adhesin T2544 using a T2544 antiserum has been shown to enhance the uptake and clearance of bacteria by host macrophages and complement-mediated lysis in mice^86^. Although antigenic variability could reduce anti-adhesion antibody efficacy, many adhesins are conserved, making them promising vaccine candidates.

## Targeting global virulence regulatory pathways

The process of colonisation and pathogenesis is governed by the ability of bacteria to perceive their external environment and the population density. This is regulated by the interconnected systems designated quorum sensing (QS), cyclic di-GMP (CdiGMP) signalling and two component signalling (TCS) systems. As these pathways play diverse roles in controlling bacterial behaviour, disrupting them represents a promising strategy to combat multiple virulence factors at once while typically not impacting bacterial growth directly (Table 2).

**Table 2:** NGAs that target the virulence regulatory pathways in bacteria.

	NGA/Active Molecule	Organism	Mode of Action	Molecular Target	in vivo*/*in vitro	Concentration Range	Reference
**Quorum Sensing Gene Expression**	Salicylic acid	*Pseudomonas aeruginosa*	Down-regulate QS systems	*lasRI* and *rhlRI*	in vitro	3.62 mM	^95^
	Trans-cinnamaldehyde	*Pseudomonas aeruginosa*	Down-regulate QS systems	*lasRI* and *rhlRI*	in vitro, in vivo	2.27 mM (95), 250 μg/mL (96)	^95, 96^
	Coumarins	*Pseudomonas aeruginosa*	Reduce expression of *rhl* and *pqs*	*rhl* and *pqs*	in vitro	1–10 mM	^98,99^
	Savirin	*Staphylococcus aureus*	Blocks Agr-regulated gene expression preventing biofilm formation	Agr QS system	in vitro, in vivo	13.5–432 μM	^102,103^
**Qourum Sensing Communica tion**	Zingerone	*Pseudomonas aeruginosa*	Binds and blocks receptor proteins LasR, RhlR and MvfR	Las, Rhl and Pqs QS systems	in vitro	100–1000 μg/mL	^180^
	Acylhomoserine lactonase	*Erwinia carotovora, Gram-negative species*	Degrades the lactone ring present in AHL molecules	AHL molecules	in vitro	50 ng/μL (181), 500 μM–1000 μM (182)	^181,182^
	Lactonase	*Pseudomonas aeruginosa*	Quorum quenching	Signal molecules N-(3-oxododecanoyl)-Lhomoserine lactone (3-oxo-C12 HSL) and butyryl-homoserine lactone (C4 HSL)	in vitro, in vivo	2 μg/mL, 0.1-10 mg/mL	^106,183^
	Acylases	*Pseudomonas aeruginosa*	Quorum quenching	AHL molecules	in vitro, in vivo	2 mg/mL, in vitro 0–16 μM, in vivo 10 μM	^107,108^
	Oxidoreductases	*Klebsiella pneumoniae*	Quorum quenching	AHL molecules	in vitro, in vivo	0.083–83.3 μg/mL	^184^
	Garlic	*Pseudomonas aeruginosa*	Blocks Quorum sensing	LasRI QS system	in vitro, in vivo	656 mg daily capsules	^111^
	Azithromycin	*Pseudomonas aeruginosa*	Down-regulation of QS genes	Las, Rhl and Pqs QS systems	in vitro, in vivo	in vitro 2 μg/mL, in vivo 300 mg/day	^110,185^
	5-fluorouracil	*Staphylococcus aureus*	Quorum quenching, inhibits AI2 production	LuxS/AI-2	in vitro, in vivo	in vitro 0.1 μM, in vivo coated catheters	^112,186^
	Furanone C-30	*Pseudomonas aeruginosa*	Potentiator of tobramycin	LasRI QS system, *mexT*	in vitro, in vivo	200 μg/mL	^116^
**Cyclic di-GMP**	Nitric oxide	*Pseudomonas aeruginosa, Vibrio cholerae, Escherichia coli*, *Fusobacterium nucleatum*, *Serratia marcescens*, *Shewanella woodyi*, *Pseudoalteromonas*, *Vibrio fischeri*, *Staphylococcus aureus*, *Legionella pneumophila*, *Nitrosomonas europaea*, *Psudemondas putida*, *Candida albicans*, *Candida tropicalis*, and *Ulva linza*	Creates oxidative stress in the bacterial biofilm inducing dispersal and preventing motility and adhesion	NO sensors, NosP or H-NOX	in vitro, in vivo	450–500 pM (126–127), 0.1–1.5 μM (128), 0.8 ppm (188)	^126–128,187–189^
	Cahuitamycins	*Acinetobacter baumannii*	Reduces cyclic di-GMP levels	CahJ protein	in vitro, in vivo	15.6 μM	^190^
	*cis*-DA, diffusible signal factor (DSF)	*Xanthomonas campestris*, *Pseudomonas aeruginosa*, *Escherichia coli*, *Klebsiella pneumoniae*, *Staphylococcus aureus* and *Candida albicans*	Modulation of cyclic-di-GMP levels and dispersal of biofilms	*rpf* gene cluster	in vitro	2.5 nM	^191,192^
	BdcA	*Escherichia coli*, *Pseudomonas aeruginosa*, *Pseudomonas fluorescens*, and *Rhizobium meliloti*	Sequesters unbound cyclic di-GMP, reducing the available concentration of cyclic di-GMP	Cyclic di-GMP	in vitro	8–10 μM	^193–196^
	Analogs of cyclic dinucleotidic acid	*Synechocystis sp*	Inhibits Slr1143	Slr1143	in vitro	100 μM	^197^
	Triazole-Linked Analogues	*Pseudomonas aeruginosa, Escherichia coli*	Binds allosteric inhibitory site (I-site)	Diguanylate cyclases (DGCs)	in vitro	90 μM	^121^
	Azathioprine	*Pseudomonas aeruginosa, Escherichia coli*	Inhibits biosynthesis of cyclic di-GMP	WspR	in vitro	90 μM	^122^
	Catechol-Containing Sulfonohydrazide Compounds	*Pseudomonas aeruginosa*, *Acinetobacter baumannii*	Inhibitors of the DGC PleD	PleD	in vitro	DCI061 PleD 17.5$\pm$1.1 μM, RocR 66.3 ± 1.3 μM, DCI058 25.5 ± 1.2 μM	^123^
	Sulfasalazine and eprosartan	*Pseudomonas aeruginosa, Escherichia coli*	Bind to the GTP active site of WspR and YdeH	*Pseudomonas aeruginosa* WspR and *Escherichia coli* YdeH	in vitro	1 mM	^198^
	ABC-1 and 2-[(4chlorobenzyl)thio]-5methoxy-1H-benzimidazole (ABC-2)	*Vibrio cholerae*	Reduce the intracellular concentration of c-di-GMP	VC1673*-lux*	in vitro	Up to 100 μM	^124^
	ABC-1	*Pseudomonas aeruginosa, Klebsiella pneumoniae, Erwinia amylovora, Shigella boydii*, and *Staphylococcus aureus*	Reduce the intracellular concentration of c-di-GMP	*Pseudomonas aeruginosa* WspR and *Escherichia coli* YdeH	in vitro	Up to 100 μM	^124^
	2'-F-c-di-GMP	*Pseudomonas aeruginosa*	I-site allosteric inhibition of diguanylate cyclases	I-site of DGC	in vitro	3 mM	^120^
**Two Component Systems**	Radicicol	*Salmonella*	Down-regulates expression of *lasRI* and *rhlRI* QS systems	PhoQ	in vitro	10 mM	^199^
	Mucin glycans	*Pseudomonas aeruginosa*	Binds to TCS GacS-GacA, downregulating the T6SS	GacS-GacA	in vitro	0.5% w/v	^137^
	Maprotiline	*Francisella novicida*	Interaction with the periplasmic sensor domain of QseC	QseC	in vitro, in vivo	0.01 μM–100 μM	^133^
	LED209	*Salmonella enterica serovar typhimurium* and *Francisella tularensis*	QseC inhibitor	QseC	in vitro, in vivo	1 ng/mL (134) 5 pM (135)	^134,135^
	Xanthoangelol B	*Staphylococcus aureus*	Bind to SaeS sensor component of TCS	SaeS	in vitro, in vivo	2.1 μM	^136^

### Disrupting QS

QS systems are utilised by bacteria as a form of communication to coordinate community phenotypes such as biofilm formation^87–89^. There are three main QS systems. Gram-positive bacteria use specific signalling peptides such as autoinducing peptides (AIPs), and Gram-negative bacteria use N-acylhomoserine lactones (AHLs). Autoinducer-2 (AI-2) is a furanosyl borate diester and is a non-pathogen specific QS molecule. It can facilitate interspecies communication as it is utilised by both Gram-positive and Gram-negative species^90^. The concentration of autoinducer increases as bacteria grow until a threshold is met. When this point is reached, the cognate response regulators are activated through autoinducer binding and are able to bind to the promoter regions of their target genes, modulating their expression^91,92^. Given the prevalence of QS systems among pathogens and the key role they play in virulence, targeting QS has become one of the most well-studied strategies for the development of NGAs.

The entire QS regulatory system has been shown to be vulnerable to targeted disruption resulting in virulence attenuation. QS inhibitors can inhibit the expression of components of the QS system or disrupt the interaction between the autoinducer and their cognate receptor proteins. By doing so, these inhibitors can block cell-to-cell communication, biofilm formation and virulence factor production^93,94^. Salicylic acid and trans-cinnamaldehyde have both been shown to effectively down-regulate the *las* (LasRI) and *rhl* (RhlIR) QS systems in *P. aeruginosa,* in vitro^95,96^. The specificity of these effects however vary from species to species, with salicylic acid having been shown to stabilise *S. aureus* biofilms, preventing dispersal^97^. Several classes of coumarins have also been identified as potent inhibitors of AHL based QS systems, with the simple coumarin molecule being shown to reduce expression of the *las*, *rhl* and *pqs* QS systems in *P. aeruginosa* and as a result decrease biofilm formation, motility, Type III Secretion System (T3SS) and phenazine production^98,99^. This activity has been shown to extend to several clinically relevant Gram-positive and Gram-negative bacteria, although the precise mechanism of QS inhibition remains to be uncovered. However, it is worth noting that molecular docking suggests direct interactions with autoinducer synthases^100,101^. A small-molecule virulence inhibitor, savirin, has been shown to inhibit the Agr QS system in *S. aureus* by binding to AgrA, preventing its ability to bind to target promoters and ultimately blocking Agr-regulated gene expression, critically at concentrations that do not impact growth^102^. This molecule has demonstrated efficacy in animal models of biofilm-related *S. aureus* skin, subcutaneous and prosthetic joint infections by rending the bacteria more susceptible to clearance by skin host defence mechanisms^102,103^.

Bacteria often compete with other species for the same ecological niche in the natural environment, one strategy that has evolved to increase fitness in this scenario is to disrupt communication between members of the competitor species. The extracellular hydrolysis of autoinducer molecules lowers their local concentration in a process known as quorum quenching (QQ), triggering biofilm dispersal and reduced virulence factor production. QQ enzymes include lactonases, acylases and oxidoreductases and predominantly target AHLs^104^. Intriguingly, some eukaryotes have been shown to encode QQ enzymes with the capacity to disrupt virulence, in either an example of chance functional promiscuity or perhaps an evolved antivirulence strategy^105^. Several QQ enzymes have been purified and shown to exhibit potent antivirulence potential against *P. aeruginosa* in a range of in vivo infection models such as a rat pneumonia model, mouse burn wound model and a mouse pulmonary infection model. The diversity of formulation and delivery of these enzymes also demonstrates their clinical potential with aerosolization, direct application and incorporation into hydrogels and coatings all proving effective delivery mechanisms^106–109^.

In the early 21^st^ century, there was considerable excitement about the clinical potential of strategies to target QS, with several pilot clinical trials taking place^110–112^. However, despite the results of these trials being largely positive, the clinical momentum has slowed. This may be impacted due to the emerging evidence that one of the most well studied and targeted QS pathways, the LasRI QS system in *P. aeruginosa*, is prone to mutations causing loss of function. This indicates that targeting specific QS systems in infection scenarios may not be as effective as originally hoped or as observed in lab adapted strains^113–115^. There has also been some evidence that resistance can evolve to certain classes of QS inhibitor such as furanones^116^. However, despite these clear limitations, there is still considerable therapeutic promise in targeting QS as a means to tackle the rise in MDR infections.

### Blocking CdiGMP signalling

CdiGMP is a secondary messenger molecule produced by diguanylate cyclases (DGCs) and utilised by bacteria to control a broad range of cellular processes, such as biofilm formation, adhesion, motility and virulence^117,118^. When CdiGMP binds to effector proteins, it has the potential to influence activity, stability, subcellular location, and the proteins’ ability to interact with other proteins^117^. High levels of CdiGMP are a known trigger of biofilm formation within numerous bacterial species, making approaches to disrupt the regulatory influence of CdiGMP an attractive target for the development of NGAs^119^. Approaches to disrupt CdiGMP signalling and as a result limit pathogenic potential include the use of synthetic CdiGMP analogs to jam the signalling cascade^120,121^, disrupting intracellular nucleotide pools^122^ and the use of DGC active site inhibitors^81,123,124^. One of the most developed strategies, however, is the use of the nitric oxide to modulate the activity of phosphodiesterases, the enzymes that breakdown intracellular CdiGMP. Exposure to NO has been shown to breakdown and reduce CdiGMP levels by activating CdiGMP-specific phosphodiesterases in bacteria^25,125–127^. Low-dose nitric oxide was also found to cause a significant reduction in *P. aeruginosa* biofilm aggregates, in CF patients, highlighting the clinical potential of this approach^128^. As this is an eubacterial secondary messenger, the risks for off target effects needs robust consideration when developing NGAs to target this signalling pathway.

### Inhibiting TCS

TCS is utilised by bacteria to sense and respond to changes in the surrounding environment. These systems are critical for bacteria to quickly recognise and adapt to different environmental conditions or threats such as changes in temperature, pH, or nutrient availability^129^. TCSs are typically composed of two proteins, a sensor kinase, and a cognate response regulator^130^. The sensor kinase contains a sensor domain that is sensitive to specific environmental signals and undergoes conformational change that activates the kinase domain of the protein. This change then results in the phosphorylation of the histidine residue within the protein. This phosphorylated sensor kinase can then go on to transfer its phosphate group to the response regulator, which contains a DNA-binding domain. Phosphorylation of the response regulator results in a conformational change, which allows for the binding of specific promoter DNA sequences that can then result in the activation or repression of the transcriptional targets^131,132^. Maprotiline, an FDA-approved tetracyclic antidepressant drug, reduces *Francisella novicida* biofilm formation through a predicted interaction with the periplasmic sensor domain of histidine kinase, QseC. Treatment of mice infected with *F. novicida* was shown to improve survival and delay disease onset^133^. Another QseC inhibitor, the small molecule LED209, was shown to inhibit QseC ligand binding and the resulting autophosphorylation without impacting bacterial viability but critically disabling several virulence mechanisms. It has demonstrated promising efficacy against *S. typhimurium* and *F. tularensis* in mouse infection models^134,135^. Xanthoangelol B, a prenylated chalcone from the plant *Angelica keiskei*, along with structural derivatives have been shown to directly bind to SaeS, the sensor component of the TCS SaeRS, a major regulator of virulence factor expression in *S. aureus*^136^. Mucin glycans have also recently been demonstrated to directly inhibit the TCS GacS-GacA in *P. aeruginosa* by binding to the antagonistic RetS sensor kinase. This then causes the down regulation of the type 6 secretion system (T6SS) which is associated with bacterial killing^137^. Despite their role in responding to stimuli, TCS remain a comparatively understudied area for the development of NGAs perhaps due to the essentiality of certain two-component sensors or the potential for host toxicity due to the similarity between kinase domains among bacteria and eukaryotes^138,139^.

## Targeting toxins

Targeting bacterial toxin functionality as a means to limit disease has a long and established history. This approach traces back to the late 19^th^ century when von Behring and Kitasato developed antibody-based antitoxins for *Corynebacterium diphtheriae* toxin and *Clostridium tetani* toxin. Their ground-breaking work earned the Nobel Prize for Medicine in 1901^140^. Over the years, antibody-based antitoxins have made significant progress and have since made their way to clinic. Notably, human monoclonal antibodies targeting *Clostridium difficile* toxin A and B (actoxumab and bezlotoxumab respectively) having been shown to significantly reduce *C. difficile* recurrence in several animal models at non-toxic concentrations^141,142^ and in human clinical trials^143,144^. However, in phase III clinical trials, only bezlotoxumab alone was shown to reduce *C difficile* recurrence and as a result was given FDA approval in 2016^145^. Toxin targeting antibodies have also shown considerable therapeutic promise against other pathogens such as *P. aeruginosa*, *S. aureus* and *Salmonella spp*^146–150^.

Consequently, secretion systems can be targeted with NGAs at the level of component expression, apparatus assembly, toxin localisation or toxin activity (Table 3). In *V. cholera*, the transcription of cholera toxin and the toxin coregulated pilus are both regulated by the transcriptional activator ToxT. Through high-throughput screening, the compound 4-[N- (1,8-naphthalimide)]-nbutyric acid (Virstatin) was found to prevent ToxT dimerisation, which is required for promoter binding. In turn, this inhibition blocks the production of the cholera toxin without affecting the growth of the bacteria^151,152^. The plant phenolic compounds TS027 and TS103 have been shown to impact the regulation of the GacSA-RsmYZ-RsmA-ExsA regulatory pathway in *P. aeruginosa* which mediates the expression of the toxins of the T3SS^153^. Salicylidene acylhydrazides have been shown to interfere with the regulation of the T3SS by altering iron availability in bacteria such as *Yersinia pseudotuberculosis* and *Chlamydia trachomatis*^154^. Since this initial discovery, the salicylidene acylhydrazide INP0341 has gone on to show considerable therapeutic promise in corneal, burn and vaginal in vivo models of *C. difficile, P. aeruginosa, S. typhimurium, Shigella, C. trachomatis, E. coli* infections^142,154–161^.

**Table 3:** NGAs that target toxin production and secretion in bacteria.

	NGA/Active Molecule	Organism	Mode of Action	Molecular Target	in vivo*/*in vitro	Reference
**Toxins**	Daio-kanzo-to	*Vibrio cholerae*	Inhibit the function of cholerae toxin	Cholerae toxin	in vitro, in vivo	^200^
	Apple polyphenol extract (APE)	*Vibrio cholerae*	Inhibit the ADP-ribosylation activity	Cholera toxin A1 fragment	in vitro, in vivo	^201^
	4-[N- (1,8-naphthalimide)]-n-butyric acid (virstatin)	*Vibrio cholerae*	Post-transcriptionally inhibits ToxT, blocking the production of the cholerae toxin	ToxT	in vitro, in vivo	^151,152^
	MDT (3- (methylthio)-1,4-diphenyl1H-1,2,4-triazolium bromide)	*Escherichia coli*	Formation of a complex that prevents the assemble of the toxin	Entry point of a single B pentamer	in vitro, in vivo	^202–204^
	TS027 and TS103	*Pseudomonas aeruginosa*	Interfere with the regulation of the GacSA-RsmYZ-RsmA-ExsA regulatory pathway reducing expression of the *exoS* toxin	GacSA-RsmYZ-RsmA-ExsA	in vitro, in vivo	^205^
	Salicylidene acylhydrazides	*Clostridium difficile, Pseudomonas aeruginosa, Salmonella enterica serovar typhimurium, Shigella, Chlamydia trachomatis, Escherichia coli*	Interferes with T3SS	T3SS	in vitro	^142,153–160^
	Resveratrol tetramer-hopeaphenol	*Yersinia pseudotuberculosis*	Inhibits the secretion of *Yersinia* outer membrane proteins by binding to the T3SS	Not identified	in vitro	^206^
	Bezlotoxumab	*Clostridium difficile*	Neutralising antibody against toxin A and B	Toxin A and B	in vitro, in vivo	^145,207^
	Fluorothiazinon	*Chlamydia* spp., *Pseudomonas aeruginosa*, and *Salmonella*	Not identified	T3SS	in vitro, in vivo	^208,209^
	Anethole	*Vibrio cholerae*	Down-regulation of cholera toxin	cAMP receptor protein	in vitro, in vivo	^210^
	Pseudolipasin A	*Pseudomonas aeruginosa*	Inhibits T3SS toxin ExoU cellular toxicity	T3SS toxin ExoU	in vitro	^165^
	Bafilomycin A1	*Clostridium difficile*	v-ATPase inhibitor	TcdB	in vitro	^166,167^
	Endogenous antibodies (eAbs)	*Clostridium difficile*	Binds and neutralises *Clostridium difficile* toxin B	*Clostridium difficile* toxin B	in vitro, in vivo	^211^
	4-bromobenzaldehyde N-(2,6dimethylphenyl) semicarbazone (EGA)	*Clostridium botulinum*	Inhibits several botulinum neurotoxins	botulinum neurotoxins	in vitro, in vivo	^168^
	Tamoxifen	*Escherichia coli*	Inhibitor of STx2 trafficking	Ribosome	in vitro, in vivo	^169,170^
	Tanshinones	*Pseudomonas aeruginosa*	Prevents T3SS needle biogenesis	T3SS needle	in vitro	^161^
	Suvratoxumab	*Staphylococcus aureus*	Binds and neutralises α toxin	α toxin	in vitro, in vivo	^212^
	Phenoxyacetamide MBX 1641	*Yersinia pestis* and *Pseudomonas aeruginosa*	Binds to PscF component of T3SS needle, preventing assembly	PscF	in vitro	^162–164^
	Raxibacumab	*Bacillus anthracis*	Binds to PA, blocking its binding to host cell receptors	Protective antigen (PA) in anthrax	in vitro, in vivo	^213,214^
	Anthrax immune globulin (AIG)	*Bacillus anthracis*	Binds to PA, blocking its binding to host cell receptors	Protective antigen (PA) in anthrax	in vitro, in vivo	^214^
	ETI-204	*Bacillus anthracis*	Binds to PA, blocking its binding to host cell receptors	Protective antigen (PA) in anthrax	in vitro, in vivo	^214^
	Obiltoxaximab	*Bacillus anthracis*	Binds to PA, blocking its binding to host cell receptors	Protective antigen (PA) in anthrax	in vitro, in vivo	^215^

Tanshinones, herbal compounds commonly used in traditional Chinese medicine, have been shown to bind directly to components of the *P. aeruginosa* T3SS needle, preventing needle biogenesis^161^. Several tanshinones have now been shown to prevent the secretion of T3SS associated toxins to macrophages in vitro and demonstrated efficacy in a murine model of acute pneumonia^162^. Phenoxyacetamide MBX 1641 was found to bind to the PscF component of the T3SS needle protein in *Yersinia pestis* and *P. aeruginosa*, preventing assembly. This inhibitor was found to decrease T3SS mediated cytotoxicity against eukaryotic cells^163–165^. Several small molecule inhibitors of toxin function have been identified and characterised with promising clinical potential. Pseudolipasin A was shown to be an inhibitor of the *P. aeruginosa* T3SS toxin, ExoU. This inhibitory activity is predicted to occur through the direct binding of this compound to the ExoU catalytic domain^166^.

An alternative strategy to overcome toxin-mediated virulence is to disrupt the eukaryotic intracellular trafficking of the toxin to its target. Endosome-lysosome acidification is required for the delivery of the *C. difficile* toxin, TcdB, across the endosomal membrane. This can be effectively inhibited by the general v-ATPase inhibitor bafilomycin A1 as well as several other compounds with lysosomotropic features including the antimalarial drug quinacrine. Preventing the transition of TcdB across the endosomal membrane was sufficient to inhibit TcdB induced cell rounding^167,168^. The intracellular trafficking of several botulinum neurotoxins has been shown to be inhibited by 4-bromobenzaldehyde N-(2,6-dimethylphenyl) semicarbazone (EGA) effectively reducing neurotoxicity in mouse models^168^. The cellular toxicity of Shigatoxins STx, STx1, and STx2 is dependent on their retrograde trafficking to their cytosolic target, ribosomes. Several promising compounds have been identified that can disrupt this trafficking and limit toxin activity, including the FDA approved breast cancer chemotherapeutic tamoxifen, which was shown to be a potent inhibitor of STx2 trafficking. Mouse toxicity studies demonstrated that human-approved doses of 10 μM of tamoxifen could significantly improve survival after exposure to a lethal amount of STx1 or STx2^169–171^.

While targeting toxin production may be an effective mechanism to limit acute infection, there is evidence that as a chronic infection develops, toxin production declines, with examples of T3SS inactivating mutations in *P. aeruginosa* chronic CF and wound isolates^172–174^.

## Challenges and future perspectives

The urgent need for novel therapeutic strategies to tackle MDR infections is clear and NGAs represent a promising therapeutical strategy that could overcome key issues like the propensity for resistance evolution associated with traditional antibiotics (Fig. 1). The proposed weaker selection pressure of NGAs, while widely accepted, does not necessarily mean that they are resistance-proof, and the capacity for bacteria to develop mechanisms to overcome their activity is an aspect that needs to be explored in greater detail. NGAs are also expected to typically constitute less interference with mammalian signalling pathways and therefore a reduced toxicity, as they are designed to target virulence pathways that are only found in pathogens, although this obviously is not the case for all NGAs and candidates that target TCSs or host intracellular trafficking in particular need to be robustly screened for off-target effects on the host. There is also the potential that although targeted towards specific pathogens, that NGAs could disrupt the behaviour of commensals within our microbiome, with for example disrupting CdiGMP potentially impacting interspecies competition and the biofilm- forming capacity of commensals within the gut microbiome.

Plant extracts are considered a rich reservoir for bioactive chemicals with high therapeutic potential and have proven to be a rich source of NGA leads. Phytochemicals occupy a chemical space with a far greater structural diversity than synthetic compound libraries and tend to be more ‘drug-like’, with superior ADME/T (absorption, distribution, metabolism, excretion and toxicity) properties. This is due to the evolutionary pressures faced by plants who have endured millennia of intensive selective pressure to develop small molecules that target specific pathways in bacteria to prevent colonisation^175^. However, a key limitation to the potential of phytochemicals as NGAs is the inherent difficulty in identifying the active molecule within a bioactive plant extract and understanding the specific cellular targets and underlying mechanisms of action, information often necessary for the pre-clinical development of NGAs. This highlights the potential of repurposing previously approved drugs as NGAs, with numerous examples having already been described of drugs having off target antivirulence effects on bacteria^133,170,171^. Similar potential has been seen with dietary compounds, with artificial sweeteners for example having been recently shown to limit the pathogenicity of several MDR pathogens when used at sub-inhibitory concentrations^176^. To effectivity stem the tide of MDR pathogens sweeping through our hospitals, it is essential we continue to develop multiple different approaches to tackle these pathogens. Targeting virulence rather than viability is an alternative approach that holds significant therapeutic potential and is likely to have increased clinical importance in the coming years.
